# Host Defense Effectors Expressed by Hemocytes Shape the Bacterial Microbiota From the Scallop Hemolymph

**DOI:** 10.3389/fimmu.2020.599625

**Published:** 2020-11-12

**Authors:** Roxana González, Ana Teresa Gonçalves, Rodrigo Rojas, Katherina Brokordt, Rafael Diego Rosa, Paulina Schmitt

**Affiliations:** ^1^Doctorado en Acuicultura. Programa Cooperativo Universidad de Chile, Universidad Católica del Norte, Pontificia Universidad Católica de Valparaíso, Valparaiso, Chile; ^2^Laboratorio de Genética e Inmunología Molecular, Facultad de Ciencias, Instituto de Biología, Pontificia Universidad Católica de Valparaíso, Valparaíso, Chile; ^3^GreenCoLab, Centro de Ciências do Mar, Universidade do Algarve, Faro, Portugal; ^4^Laboratorio de Patobiología Acuática, Departamento de Acuicultura, Universidad Católica del Norte, Coquimbo, Chile; ^5^Laboratorio de Fisiología Marina (FIGEMA), Departamento de Acuicultura, Facultad de Ciencias del Mar, Universidad Católica del Norte, Antofagasta, Chile; ^6^Laboratory of Immunology Applied to Aquaculture, Department of Cell Biology, Embryology and Genetics, Federal University of Santa Catarina, Florianópolis, Brazil

**Keywords:** host-microbiota interactions, invertebrate immunity, mollusk, *Vibrio*, host defense effectors, big defensin, bactericidal/permeability-increasing protein

## Abstract

The interaction between host immune response and the associated microbiota has recently become a fundamental aspect of vertebrate and invertebrate animal health. This interaction allows the specific association of microbial communities, which participate in a variety of processes in the host including protection against pathogens. Marine aquatic invertebrates such as scallops are also colonized by diverse microbial communities. Scallops remain healthy most of the time, and in general, only a few species are fatally affected on adult stage by viral and bacterial pathogens. Still, high mortalities at larval stages are widely reported and they are associated with pathogenic *Vibrio*. Thus, to give new insights into the interaction between scallop immune response and its associated microbiota, we assessed the involvement of two host antimicrobial effectors in shaping the abundances of bacterial communities present in the scallop *Argopecten purpuratus* hemolymph. To do this, we first characterized the microbiota composition in the hemolymph from non-stimulated scallops, finding both common and distinct bacterial communities dominated by the Proteobacteria, Spirochaetes and Bacteroidetes phyla. Next, we identified dynamic shifts of certain bacterial communities in the scallop hemolymph along immune response progression, where host antimicrobial effectors were expressed at basal level and early induced after a bacterial challenge. Finally, the transcript silencing of the antimicrobial peptide big defensin *ApBD1* and the bactericidal/permeability-increasing protein *ApLBP/BPI1* by RNA interference led to an imbalance of target bacterial groups from scallop hemolymph. Specifically, a significant increase in the class Gammaproteobacteria and the proliferation of *Vibrio* spp. was observed in scallops silenced for each antimicrobial. Overall, our results strongly suggest that scallop antimicrobial peptides and proteins are implicated in the maintenance of microbial homeostasis and are key molecules in orchestrating host-microbiota interactions. This new evidence depicts the delicate balance that exists between the immune response of *A. purpuratus* and the hemolymph microbiota.

## Introduction

Scallops are a cosmopolitan family of bivalve mollusks. In recent years, interest in scallop immunity has increased due to its economic importance for aquaculture, and because the group occupies a key position within the phylogeny and evolution of the animal kingdom (1). Bivalves have a semi-open circulatory system where the hemolymph, the analogue of vertebrate blood, and the organs are constantly exposed to high and diverse bacterial loads (~10^6^ cfu/ml) (2). Still, scallops remain healthy most of the time, and in general, only a few species are fatally affected on adult stage by viral and bacterial pathogens (3–5).

Like the rest of invertebrates, scallops possess only innate immune mechanisms of defense, where cellular and humoral components act in a coordinated and synergistic manner (6). Hemocytes, the immunocompetent cells, circulate within hemolymph and infiltrate tissues, constantly producing a great diversity of antimicrobial effectors (7). When scallops are exposed to microorganisms, many of these effectors are released by the hemocytes to the hemolymph or into infiltrated tissues, where they classically inactivate or destroy foreign invaders (8, 9). Antimicrobial peptides from the big defensin family such as *ApBD1* (10–12), hydrolytic enzymes (13, 14), lipopolysaccharide binding/bactericidal permeability increasing proteins (LBP/BPIs) (15), and an antimicrobial peptide derived from histone (HDAP) (16) have been described to be expressed by scallop hemocytes. Furthermore, the recent genome sequencing and *de novo* assemblies of transcriptomes from several scallop species suggests the existence of many other gene candidates for antimicrobial effectors (17–20).

In recent years, the interaction between the immune response and the microbiota has become a fundamental aspect of animal health (21, 22). This interaction allows the conformation of the microbial communities, which participate in a variety of processes in the host species, such as stimulating the immune system (23, 24), nutrition (25), protection against pathogens (26–28), among others. In higher vertebrates as well as in model and non-model invertebrates, a delicate balance exists between the basal expression of immune effectors and the microbial host associations (22, 29, 30). If this balance is disrupted, pathogenic microorganisms can proliferate causing damage and host mortality (22, 31). In the oyster *Crassostrea gigas*, microbiota destabilization caused by viral infection and subsequently host immunosuppression ended in bacteremia and host death (32). In addition, differential basal expression of immune genes has been related with oyster resistance to this polymicrobial disease (33). Recently, changes in the structure and diversity of bacterial microbiota associated with scallops has been identified during the immune response of *Argopecten purpuratus* in field (34). Overall, these results suggest that functional interaction between host immune effectors and associated microbiota could also exist in pectinids.

To give new insights into the scallop-microbiota interactions, we assessed the involvement of two host antimicrobial effectors, *ApBD1* and *ApLBP/BPI1* in shaping the hemolymph scallop microbiota. We first characterized the structure and relative abundances of bacterial communities present in hemolymph from non-stimulated scallops. Then, we identified significant shifts and restorations of specific bacterial groups along immune response progression. In here, antimicrobial effectors were expressed at basal levels and early induced after bacterial challenges. Finally, we investigated the effect of silencing the antimicrobials *ApBD1* and *ApLBP/BPI1* expressions in the bacterial abundances from non-stimulated scallops. Results obtained here suggest that antimicrobial peptides and proteins effectors are implicated in the maintenance of microbial homeostasis and are key molecules in orchestrating host-microbiota interactions in scallops.

## Material and Methods

### Scallop Maintenance, Experimental Challenges, and Sample Collection

US National Research Council guidelines for the care and use of laboratory animals were strictly followed during this research (35). Adult *Argopecten purpuratus* (n = 123; 60 mm length) were obtained from the aquaculture concession of the Universidad Católica del Norte (UCN) located at Tongoy Bay, Coquimbo, Chile. Scallops were transferred to the Central Laboratory of Marine Cultures on the premises of UCN, acclimatized and kept in 200-L tanks for two weeks prior to each experiment. Animals were constantly maintained with aeration and replacement of filtered water at 16°C. Animals were fed daily with a mixture of microalgae (50% *Isochrysis galbana* and 50% *Nannochloris* spp.; 6x10^6^ cells/ml/day). *Vibrio splendidus* bacterial strain VPAP18 was used in challenge experiments (36). The bacteria were cultivated in Trypticase Soy Broth (Difco) supplemented with 2% NaCl at 18°C for 24 h. Subsequently, the bacterial suspension was inactivated for 2 h at 90°C and centrifuged at 12,000 × g for 10 min. The obtained pellet was washed twice with 0.22 µm microfiltered sterile seawater, and bacterial solution concentration was adjusted to 1 ×10^8^ cfu/ml. *V. splendidus* VPAP18 was heat inactivated and washed to eliminate microbe-microbe interaction between *V. splendidus* VPAP18 and scallop microbiota.

Two independent scallop immune challenges were performed, both including the following experimental conditions: (i) *Vibrio*-injected scallops (5×10^6^ cells in 50 µl), (ii) seawater-injected (SW) scallops (50 µl) and (iii) non-stimulated scallops. Samples were obtained after 12, 24, 48, 72 and 168 h. For the RNAi experiment [RNAi, ribonucleic acid (RNA) interference], experimental conditions considered scallops injected with 20 µg in 100 µl of: (i) *Ap*BD1-dsRNA (dsRNA, double-stranded ribonucleic acid), (ii) *Ap*LBP/BPI1-dsRNA and (iii) GFP-dsRNA (green fluorescent protein). Samples were obtained after 48 h. Scallop sample size for each experiment is shown in **Figure S1**. All animals were injected with sterile syringes (25G × ⅝) into the adductor muscle. To obtain hemocytes, 2 ml of hemolymph per individual were extracted and centrifuged at 800 × g for 10 min at 4°C to separate cells from plasma. Then, cells were lysed in Trizol^®^ reagent (ThermoFisher) at 4°C and stored at -80°C until total RNA purification. To obtain hemolymph bacteria, 2 ml of hemolymph were deep-frozen in liquid nitrogen and stored at -80°C for subsequent extraction of genomic DNA (gDNA).

### RNA Extraction and Gene Expression Analysis by RT-qPCR

Total hemocyte RNA was extracted using Trizol^®^ reagent (ThermoFisher) according to manufacturer’s instructions. Genomic DNA was removed with DNase I (Ambion). The integrity of RNA was verified on agarose gels, and purity and concentration were determined on an Epoch™ microplate spectrophotometer (BioTek). The cDNA was synthesized by reverse transcription from 1 µg of total RNA, using the RevertAid First Strand cDNA Synthesis Kit (ThermoFisher), according to manufacturer’s instructions. RT-qPCR (quantitative polymerase chain reaction prior to reverse transcription) assays were performed in triplicate on a Stratagene Mx3005p Real Time PCR System thermocycler (Agilent Technologies), using the specific gene primers (**Table S1**). Amplification was performed in a final reaction volume of 20 µl composed of: 10 µl of Takyon ™ Rox SYBR^®^ MasterMix dTTP Blue (Eurogentec), 0.6 µl of each primer (10 mmol L^-1^) and 2 µl of cDNA (1:5). The amplification program consisted of an initial denaturation at 3 min at 95°C, followed by 40 cycles of 15 s at 95°C and 30 s at 60°C. Through serial dilutions of cDNA, the efficiencies of RT-qPCR were verified in the range between 95%–110% (E = 10 ^(–1/slope)^). The relative expression of the immune response genes was calculated by the Pfaffl method (37). Differences in gene expression with the control groups (SW) were verified with two-way ANOVA test and Tukey´s posterior test (*P* < 0.05).

### Genomic DNA Extraction and Deep Amplicon Sequencing of the 16S rDNA Gene

Genomic DNA (gDNA) present in *A. purpuratus* hemolymph was extracted with the Wizard Genomic DNA purification kit (Promega) according to manufacturer’s instructions. The integrity, concentration and purity of the gDNA was verified by 1% agarose gel, Qubit 3.0 (Life Technologies) and Epoch™ microplate spectrophotometer (BioTek), respectively. The 16S rDNA gene of bacterial communities was amplified and sequenced, targeting the variable regions V3-V4 (341F: 5’-CCTACGGGNGGCWGCAG-3’; 805R: 5’-159 GACTACHVGGGTATCTAATCC-3’) and using ~12.5 ng of gDNA from 5 biological replicates from each experimental condition: *Vibrio*-injected and SW-injected scallops at 48 h and 168 h, and non-stimulated scallops at 0 h. Paired-end multiplex sequencing (2×300 bp read length) was performed from individual samples by Macrogen Inc. on a MiSeq system (Illumina^®^) using the MiSeq Reagent Kit v3 according to manufacturer’s instruction. Raw sequence data are available in the SRA database BioProject PRJNA639911.

### 16S rDNA Deep Amplicon Sequencing Analysis

Raw reads were processed using QIIME2 version 2019.1 (http://qiime2.org) and developed based on standards described by (38, 39) for microbiota community evaluation. Demultiplexed paired-end reads were imported as artifacts and denoised using DADA2 (40). In this step of the analysis, quality control as well as phiX reads (commonly present in marker gene Illumina sequence data) and chimera sequence filtering were applied to ensure the retainment of only high-quality reads. Amplicon sequence variants (ASVs), a higher resolution analog to operative taxonomic units (OTUs) (41), were obtained and further processed for taxonomic assignment and diversity analysis. Taxonomy was assigned based on the Green Genes database (gg-13_8 99%) (42, 43). Features assigned to the class Chloroplast (Phylum Cyanobacteria) and Family Mitochondria (Phylum Proteobacteria) were filtered out due to their contaminant character. Samples were rarefied to the maximum depth of the sample with less sequencing depth, and rarefaction curves were plotted. Alpha diversity was evaluated using the Shannon and Simpson Diversity Index, the richness index was evaluated by Chao1 y Faith PD (Faith’s Phylogenetic Diversity) and beta diversity was evaluated by assessing weighted and unweighted UniFrac distances (44, 45). Differences in alpha diversity between groups were analyzed using the Kruskal-Wallis (pairwise) test in QIIME2. A principal coordinate analysis was performed to visualize phylogenetic beta diversity and differences between experimental groups based on the distances were assessed by PERMANOVA considering 999 permutations in QIIME2. Significant differences between relative abundances among groups were evaluated using two-way ANOVA and Tukey-Kramer tests on STAMP (Statistical Analysis of Metagenomic Profiles) (46), significant differences were only considered when *P* ≤ 0.05 and a q-value < 0.3. Bacterial groups showing abundances greater than 1% were considered for analysis.

### Absolute Quantification of 16S rDNA by qPCR and Determination of Relative Abundances of Bacterial Groups

The 16S rDNA gene of four bacterial strains belonging to the Gammaproteobacteria, Epsilonproteobacteria, Betaproteobacteria, and Firmicutes taxa (**Table S2**), plus the 16S rDNA gene from *Vibrio splendidus* VPAP18 were amplified by PCR using the 16S rDNA universal primers 16SEUBAC (**Table S1**). The 16S rDNA fragments were cloned into the pCR2.1 vector included in the TOPO TA kit following manufacturer’s instructions. Specific cloning of each 16S rDNA bacterial strain was verified by plasmid sequencing. Then, seven-serial dilutions of each plasmid, ranging from 10^10^ to 10^3^ plasmid copies/µl were prepared to construct standard curves and to obtain absolute quantifications of the 16S rDNA gene copy number of each bacterial group by qPCR. Plasmid copies were calculated by the following formula: Number of copies/µl = 6.022×10^23^ (molecules/mole) × DNA concentrations (g/µl)/Number of bases pairs × 660 Da, were 6.022×10^23^ (molecules/mole) is Avogadro´s number and 660 Da is the average weight for a single base pair.

Specific primers for the 16S rDNA of each bacterial group were previously designed and validated (**Table S1**). qPCR assays were performed in triplicate on a Stratagene Mx3005p Real Time PCR System thermocycler (Agilent Technologies), using a final reaction volume of 20 µl composed of: 10 µl of Takyon ™ Rox SYBR^®^ MasterMix dTTP Blue (Eurogentec), 0.6 µl of each primer (10mmol * L-1) and 2 µl of plasmid DNA. The amplification program consisted of an initial denaturation at 10 min at 95°C, followed by 40 cycles of 30 s at 95°C and 1 min at 60°C and dissociation curve detection. Standard curves were obtained by plotting the threshold cycle (C_q_) on the Y-axis and the natural log of concentration (copies/µl) on the X-axis. We considered the following criteria: PCR efficiency (95-110%) and correlation coefficient R^2^ (0.99), which validates the linear relation between the threshold cycle and the natural log of concentration (copies/µl). Next, 50 ng of gDNA from scallop hemolymph was analyzed by qPCR using the same settings. The copy numbers of 16S rDNA gene of each bacterial group in the sample were obtained by relating the C_q_ value to the respective standard curve. Eubacteria universal primers were used to obtain the total copies of bacterial 16S rDNA in each sample and it was considered as the 100% to calculate the relative abundance of each bacterial group. Differences in the relative abundance of the bacterial groups between treatments were verified with ANOVA two-way and the Sidak´s posterior test (*P <*0.05).

### dsRNA Synthesis for RNA Interference Assay of Antimicrobial Effectors

Sequence specific primers were designed to amplify fragments of *ApBD1* (401 bp) and *ApLBP/BPI1* (543 bp) from hemocyte cDNA as template and to add the T7 promoter sequence TAATACGACTCACTATAGGG by PCR (**Table S1**). A plasmid containing the sequence for the green fluorescent protein (GFP) gene (GenBank no. HM640279) which is not present in *A. purpuratus* was amplified with specific primers and included as a dsRNA control of specific gene silencing. The PCR products were verified on agarose gel and then purified using E.Z.N.A.^®^ Gel Extraction Kit (Omega Biotek). PCR products were confirmed by sequencing. T7 RiboMAX^®^ Express RNAi System was used to synthesize the dsRNA in accordance with manufacturer’s instructions. The quantity and quality of the RNAi were determined using a Qubit 3.0 (Life Technologies) and agarose gel electrophoresis, respectively.

### Determination of Cultivable *Vibrio* spp. in Scallop Hemolymph After Gene Silencing of Antimicrobial Effectors

Thiosulfate-citrate-bile salts-sucrose (TCBS) selective agar plates for *Vibrio* spp. (Difco) were prepared in sterile sea water (50%): distilled water 1: 1 following the manufacturer’s instructions. Aliquots of 0.1 ml and their dilutions from scallop hemolymph from the RNA interference experiment were plated separately in the selective agar plates and incubated at 25°C. Three independent aliquots from each scallop hemolymph were plated (5 scallops per experimental condition). After 24 h of plate incubation, visible colonies were counted. Then, average colony forming units (c.f.u.) number with the corresponding standard deviation were calculated for each sample. Differences in the c.f.u. number of *Vibrio* spp. between treatments were verified with Welch’s t-test (*P <*0.05).

## Results

### Characterization of Common and Variable Bacterial Communities Present in Scallop Hemolymph

The gDNA extracted from bacteria present in the hemolymph of i) heat-inactivated *Vibrio*-injected scallops, ii) SW-injected scallops, and iii) non-stimulated control scallops was analyzed by 16S rDNA deep amplicon sequencing. As a result, a total of 2,546 million bases were sequenced complying 4,229,314 joined sequences, with an average length of 300 bp, and 92.7% presented a quality score above Q20 whereas 84.6% were above Q30. After filtering, denoising and chimera removal, 3,159,967 high quality reads were obtained, ranging per individual between 108,842 and 150,048 reads, representing all the phylotypes present in the *A. purpuratus* hemolymph bacterial microbiota (**Table S3**). After filtering the rare OTUs, 478 taxonomic groups were identified in the *A. purpuratus* hemolymph. Analysis of rarefaction curves indicated that the sequencing depth was sufficient to identify all unique OTUs among all experimental groups (**Figure S2**).

**Figure 1:**
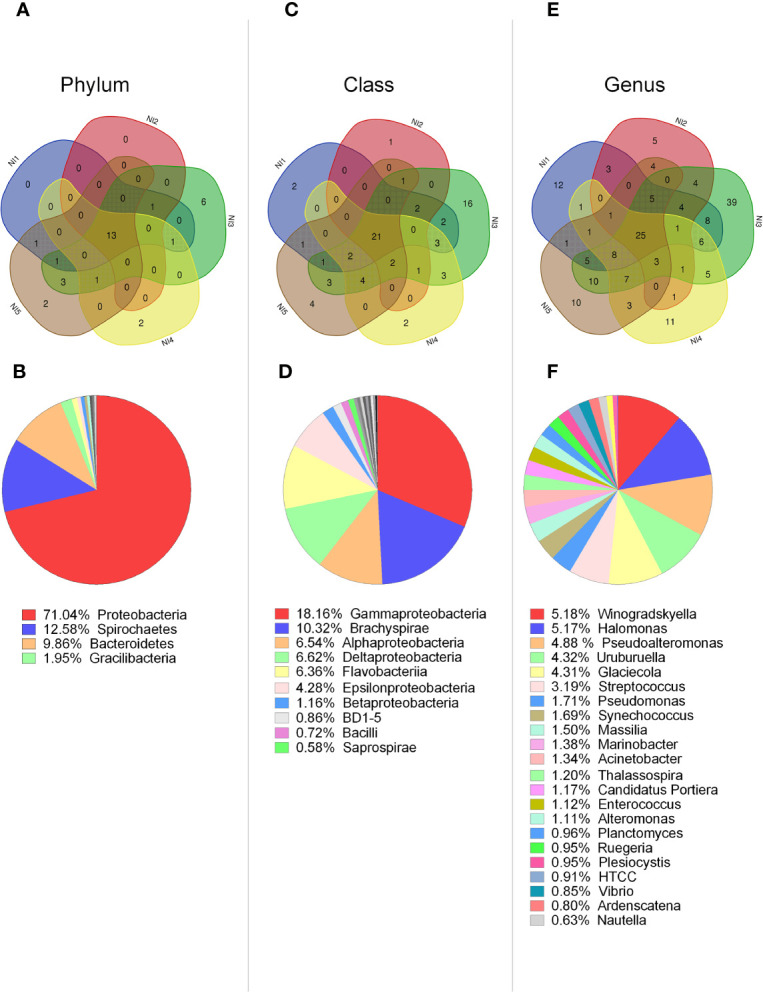
Characterization of bacterial communities present in hemolymph from single scallops determined by 16S rDNA deep amplicon sequencing. Upper panel: Venn diagrams indicate the number of common and distinct bacterial groups at phylum **(A)**, class **(C)**, and genus **(E)** levels between five non-stimulated individuals. Lower panel: Relative abundances of common bacterial groups at phylum **(B)**, class **(D)**, and genus **(F)** levels. Bacterial groups with relative abundances higher or closer to 1% are shown for each taxonomic level.

We first focused on the analysis of bacterial groups present in non-stimulated scallops to determine how variable was the scallop microbiota between control individuals. Common bacterial groups among five non-stimulated scallops were determined by Venn diagrams (**Figure 1**). From the 37 phyla identified, 35% (13 phyla) were common to the 5 individuals (**Figure 1A**). Within these 13 phyla, a 54% were predominant groups, displaying relative abundances around or higher than 1% of the total (**Figure 1B**). Those phyla were identified as Proteobacteria (71.04%), Spirochaetes (12.58%), Bacteroidetes (9.86%), Gracilibacteria (1.95%), and Firmicutes (0.92). Venn diagram analysis indicated that the number of phyla shared among the five individuals was higher than the specific phyla number found for each individual. Accordingly, two scallops lacked specific phyla, two scallops presented two specific phyla and one scallop (namely N3) displayed six particular phyla (**Figure 1A**).

From the 90 bacterial classes identified, 23% (21 classes) corresponded to common groups among all scallops (**Figure 1C**). Within these classes, 43% were predominant, displaying relative abundances higher than 1% of the total, such as: Gammaproteobacteria (18.16%), Brachyspirae (10.32%), Alphaproteobacteria (6.54%), Deltaproteobacteria (6.52%), Flavoproteobacteria (6.36%), Epsilonproteobacteria (4.28%), and Betaproteobacteria (1.16%) (**Figure 1D**). Venn diagram analysis also indicated that the number of classes shared among the five individuals was higher than the specific classes found for each individual. Therefore, four scallops presented between one to four distinct classes, and N3 scallop displayed a higher number of 16 particular classes (**Figure 1C**).

At the genus level, from the 329 bacterial genera identified in from the 5 non-stimulated scallops, 7.5% (25 genera) corresponded to common groups (**Figure 1E**). From these common genera, 88% had relative abundances higher than 1%. The most relative abundant were *Winogradskyella* (5.18%), *Halomonas* (5.17%), *Pseudoalteromonas* (4.87%), *Uruburuella* (4.32%), *Glaciecola* (4.31%), *Streptococcus* (3.19%), and *Pseudomonas* (1.71%) (**Figure 1F**). Venn diagram analysis indicated again that four scallops presented more common than particular genera, presenting between five to twelve specific genera. At this level, N3 scallop differed from the others in terms of variable groups, displaying 39 particular genera (**Figure 1E**). Overall, results suggested that control scallops share more common taxa compared to specific taxa, except for one individual that harbored an elevated number of particular groups (**Figures 1A, C, E**). In agreement, N3 individual displayed higher species richness compared to other control scallops, determined by Chao1 and Faith PD indices (**Figures S3C and D**). Similarly, the Unweighted UniFrac distance analysis confirmed that the bacterial communities from N3 individual differed from the rest of the non-stimulated scallops (**Figure S4A**).

### Shifts and Restoration of Specific Bacterial Group Abundances Are Detected in Hemolymph Along Scallop Immune Response Progression

**Figure 2:**
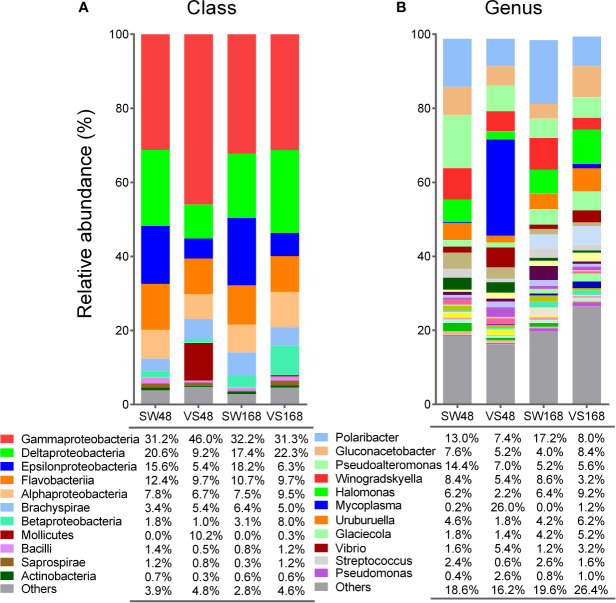
Relative abundances of bacterial groups present in scallop hemolymph after immune stimulation determined by 16S rDNA deep amplicon sequencing. Stack bar graphs indicate the average relative abundance of major bacterial groups found in *Vibrio*-injected scallops (VS) and seawater-injected scallops (SW) after 48 h and 168 h, at class **(A)** and genus **(B)** taxonomic levels. Bacterial groups with relative abundances higher or closer to 1% are shown for each taxonomic level.

A previous study identified bacterial shifts in structure and composition from whole scallop microbiota at 48 h after immune challenge (34). To determine whether imbalances in bacterial communities are sustained or restored over time after the immune response, we analyzed the bacterial composition present in the scallop hemolymph at 48 h and 168 h after challenge them. Then, the bacterial relative abundances at class and genus taxonomic levels obtained by deep amplicon sequencing analysis were compared between *Vibrio*- and SW-injected scallops (**Figure 2**). We found that the most noticeable changes on bacterial abundances between experimental conditions were observed at 48 h post challenge, at both class (**Figure 2A**) and genus (**Figure 2B**) levels. As expected, the relative abundance of Gammaproteobacteria was significantly higher in *Vibrio*-injected scallops (46%) compared to seawater scallops (31.2%) (*P*<0.05). Concomitantly, the classes Delta- and Epsilonproteobacteria displayed a lower abundance in *Vibrio*-injected (9.2% and 5.4%, respectively) when compared to SW-injected scallops (20.6% and 15.6%, respectively) (*P*<0.05). The class Mollicutes, which were found at 0.023% in SW-injected scallops, displayed an 10.2% of relative abundance in *Vibrio*-injected scallops at 48 h after immune challenge. At 168 h after immune challenge, relative abundances of Gamma-, Delta-, and Epsilonproteobacteria in *Vibrio-*injected scallops were statistically comparable to those from SW-injected scallops, suggesting a restoration of those communities after that time (**Figure 2A**). Betaproteobacteria showed no significant variation at 48 h among injected groups, though a slightly increase of this class was observed in the *Vibrio*-injected group (8%) compared to the SW-injected group (3.1%) at 168 h.

When we compared the relative abundances of bacteria at genera level, we found that *Mycoplasma* (from Mollicutes class) displayed the principal shift, although slightly non-significant (*P* = 0.065). Specifically, *Mycoplasma* increased from 0.2% in SW-injected scallops up to 26% in *Vibrio*-injected scallops at 48 h. As expected, *Vibrio* displayed significant increase in its relative abundance *in Vibrio*-injected scallops (5.4%) when compared to SW-injected scallops at 48 h (1.6%) (*P*<0.05). Remarkably, *Vibrio* genus abundance was only a fraction (11%) of total increase of Gammaproteobacteria abundance in *Vibrio*-injected scallops, revealing that the higher abundance found in this group was not only due to the immunostimulation approach. Other bacterial genera displayed shifts on their relative abundances among groups at 48 h, but still non-significant. For instance, S*treptococcus* and *Halomonas* were found in lower abundance in *Vibrio*-injected scallops (0.6% and 2.2%, respectively) when compared to SW-injected scallops at 48 h (2.4% and 6.2%, respectively). *Pseudomonas* displayed the opposite pattern at the same time point, showing a relative abundance of 2.6% in *Vibrio*-injected and 0.4% SW-injected scallops (**Figure 2B**). After 168 h of challenge, bacterial abundances at the genus level in *Vibrio-*injected scallops were also statistically similar to those from SW-injected scallops, although a higher variability in bacterial abundances were observed at this taxonomic level (**Figure 2B**). For instance, *Mycoplasma* relative abundance decreased in *Vibrio*-injected scallops after 168 h (1.2%) when compared to 48 h (26%) but it was still higher when compared to the SW-injected group which did not show any presence of this genera. Analogous variation in their relative abundances were found for *Streptococcus, Halomonas, Pseudomonas*, and *Vibrio* genera at 168 h (**Figure 2B**). Overall, our data revealed the existence of dynamic shifts of bacterial communities in the scallop hemolymph along immune response progression. Certain bacterial group shift abundances were sustained over time, while a greater proportion of bacterial groups were restored after 168 h.

Alpha diversity was evaluated using the Simpson and Shannon Diversity indices, while the species richness was evaluated by Chao1 y Faith PD indices (**Figure S3**). The Simpson index determined that *Vibrio*-injected scallops at 48 h presented more diverse bacterial communities than the other experimental conditions, specifically compared to non-stimulated scallops and SW-injected scallops at 168 h (*P*<0.05) (**Figure S3**). Shannon index presented a similar trend than the Simpson index but differences were non-significant. Similarly, the Chao1 and Faith PD richness indices suggested that *Vibrio*-injected scallops at 48 h and 168 h, respectively, tended to display a higher species richness compared to other injected scallops, although the differences were non-significant (*P*>0.05) (**Figure S3**). PCoA based on weighted (quantitative) and unweighted (qualitative) UniFrac distances suggested not distinct microbiota community among experimental conditions (**Figure S4**), with the exception of the weighted UniFrac distance between bacterial communities from SW-injected scallops at 168 h (SW168) and *Vibrio*-injected scallops at the same time (VS168) that was significant (PERMANOVA pseudo-F=2.43, P=0.023). The variance explained in the PCoA using the unweighted UniFrac was 19.08% (PC1 10.94% and PC2 8.14%) (**Figure S4A**) and the explained variance obtained using the weighted UniFrac was 54.19% (PC1 37.29% and PC2 16.91%) (**Figure S4B**).

### Significant Changes in the Bacterial Abundances From Hemolymph Occurred After the Activation of Scallop Immune Response

**Figure 3:**
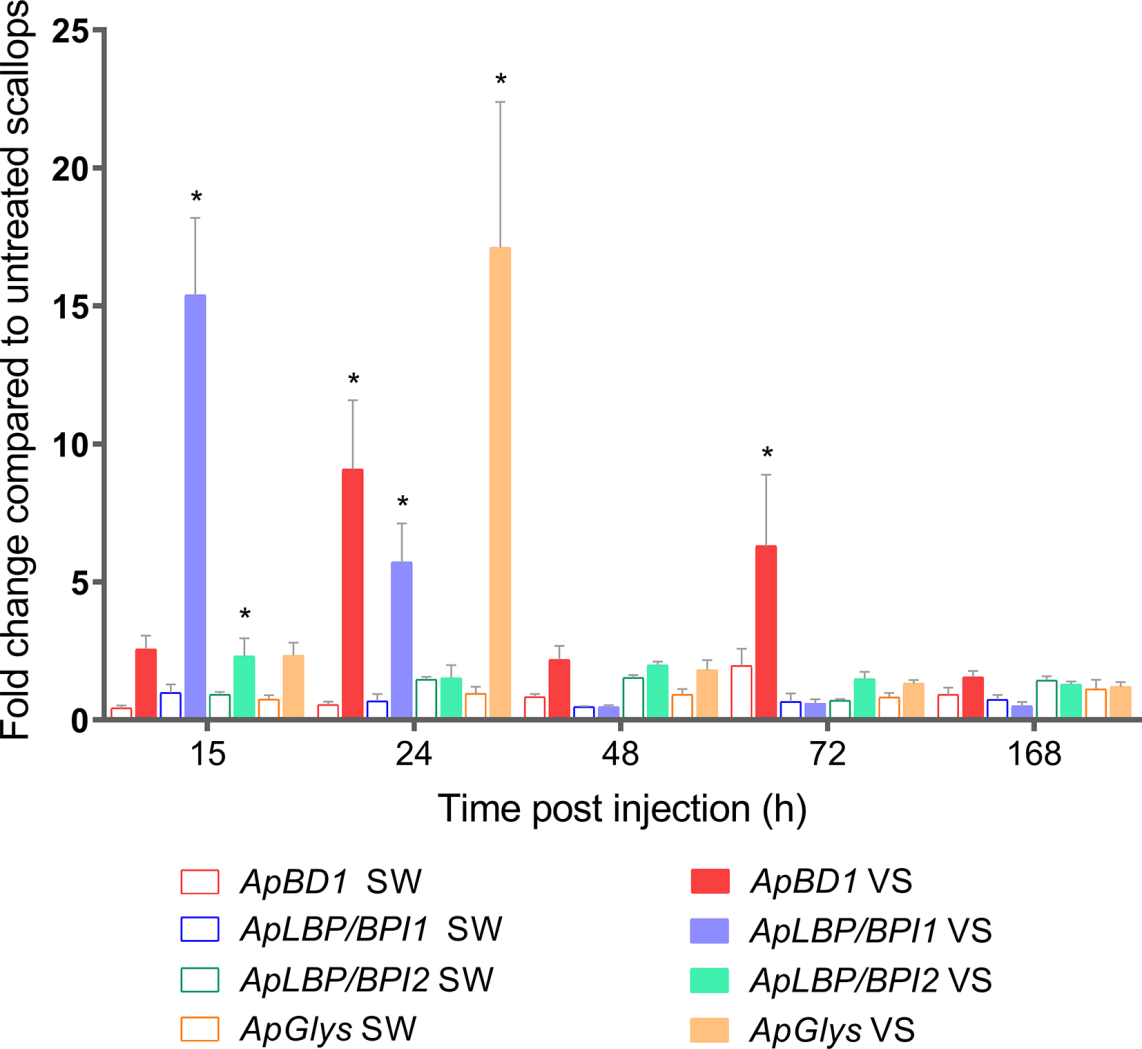
Transcript expression of *ApBD1, ApLBP/BPI1, ApLBP/BPI2*, and *ApGlys* in scallop hemocytes during immune response. Bar graphs indicate the relative expression of antimicrobial effectors genes in *Vibrio*-injected scallops (VS) and seawater-injected scallops (SW) after 12, 24, 48, 72, and 168 h. SW-injected scallops were considered as injury control condition. Relative expression was calculated using non-stimulated scallops as control group, where gene expression values were considered 1. Graphed data are represented as the mean ± ES (n = 7). Asterisks indicate significant differences compared to SW-injected scallops (*P < 0.05).

We next investigated the expression of antimicrobial effectors that could be involved in host-microbiota interaction during scallop immune response. For that, we focused on the transcription patterns of four antimicrobial effectors expressed by hemocytes from the same analyzed scallops. The relative expression of the antimicrobial peptide big defensin *ApBD1*, the antimicrobial proteins *ApLBP/BPI1* and *ApLBP/BPI2*, and the hydrolytic enzyme lysozyme *ApGlys* were assessed in all experimental conditions at 12, 24, 48, 72, and 168 h by RT-qPCR (**Figure 3**). Results showed that all antimicrobials were first overexpressed in *Vibrio*-injected scallops within the first 24 h compared to the sea water-injected control group. Thus, *ApLBP/BPI1* was overexpressed 15- and 5.7- folds at 15 and 24 h, respectively whereas *ApLBP/BPI2* was slightly overexpressed 2.3- fold at 15 h. *ApBD1* was overexpressed 9.1- and 6.3- folds s at 24 and 72 h, respectively. The *ApGlys* gene was overexpressed 17- fold at 24 h (**Figure 3**). No significant overexpression was detected for the four immune genes after 72 h post injection. Also, all genes were expressed at basal level by hemocytes of non-stimulated scallops (**Figure S5**). Overall, the significant changes observed in the scallop bacterial communities occurred after the activation of the immune response and the expression of antimicrobial effectors.

### Significant Shifts of Target Bacterial Groups Are Early Detected During Scallop Immune Response

**Figure 4:**
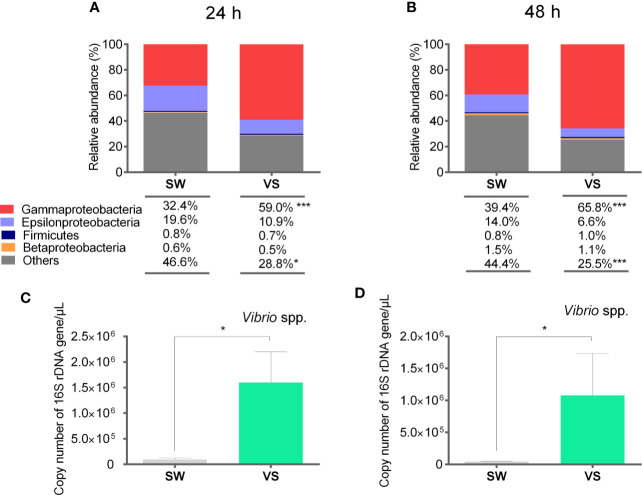
Relative abundances of target bacterial groups present in scallop hemolymph after immune stimulation determined by qPCR and absolute quantification. Stack bar graphs indicate the average relative abundance of target bacterial groups in Vibrio-injected scallops (VS) and seawater-injected scallops (SW) at 24 h **(A)** and 48 h **(B)** after challenge. Number of total copies of specific bacterial 16S rDNA gene for *Vibrio* spp. at 24 h **(C)** and 48 h **(D)** after challenge are represented as the mean ± SE, and asterisks indicate significant differences compared to SW-injected scallops (***P < 0.005; *P < 0.05).

In an attempt to assess the changes of target bacterial groups in scallop hemolymph during the expression of immune effectors, we determined their relative abundances by qPCR and absolute quantification of 16S rDNA. To do this, a second immune challenge was performed and the significant overexpression of antimicrobial effectors in scallop hemocytes was confirmed at 24 h post challenge (**Figure S6**). Then, we examined the relative abundances of Gamma-, Epsilon-, and Betaproteobacteria, Firmicutes, as well as the specific quantification of *Vibrio* spp. from scallop hemolymph at 24 and 48 h after challenge (**Figure 4**). Results showed that significant shifts in bacterial relative abundances could be detected in scallop hemolymph as early as 24 h after challenge (**Figure 4A**). Furthermore, the relative abundances of Gamma-, Epsilon- and Betaproteobacteria, and Firmicutes were similar in *Vibrio*-injected scallops compared to SW-injected scallops at both 24 h and 48 h (**Figures 4A, B**). We observed a significant increase in the Gammaproteobacteria (*P*<0.001) and a significant decrease in undetermined bacterial groups (others) (*P*<0.05) in *Vibrio*-injected scallops at both time points. Still, Gammaproteobacteria exhibited a higher relative abundance at 48 h (65.8%) compared to 24 h (59%) after *Vibrio* injection. Betaproteobacteria and Firmicutes relative abundances did not vary between groups or time points. Epsilonproteobacteria abundance decreased in *Vibrio*-injected scallops at 24 h and 48 h, still those changes were non-significant (*P*>0.05). When comparing data obtained by absolute quantification of 16S rDNA and the deep amplicon sequencing from samples at 48 h, results showed a consistent shift trend for Epsilon-, Beta-, and Gammaproteobacteria but not for Firmicutes.

We further determined the number of total copies of specific bacterial 16S rDNA gene of the genus *Vibrio* to explain the contribution of the *Vibrio* injection to the Gammaproteobacteria abundance observed in scallop hemolymph (**Figures 4C, D**). As determined by the deep amplicon sequencing analysis, the increase *Vibrio* spp. was only a fraction of total Gammaproteobacteria relative abundance. Indeed, in terms of relative abundance, *Vibrio* spp. correspond to the 50% and 16% of total Gammaproteobacteria at 24 and 48 h respectively. Thus, other bacteria belonging to the Gammaproteobacteria class increased their abundances in *Vibrio*-injected scallops.

### Gene Silencing of ApBD1 and ApLBP/BPI1 by RNAi in Non-Stimulated Scallops Leads to an Imbalance of Bacterial Groups From the Hemolymph Which Is Associated With *Vibrio* spp. Proliferation

**Figure 5:**
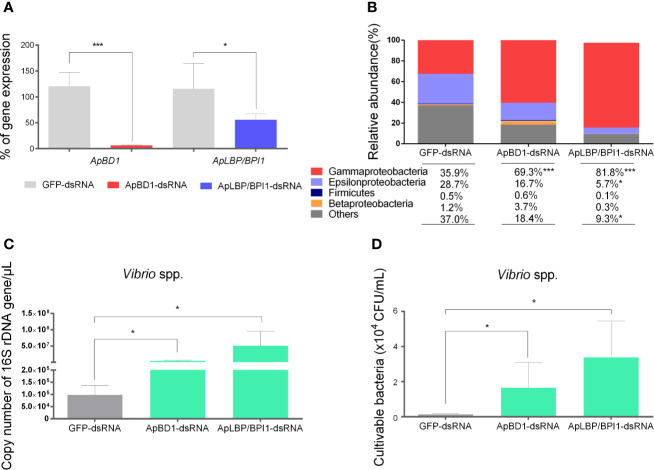
Effect of the silencing of *ApBD1* and *ApLBP/BPI1* expression in scallop by RNAi on the relative abundances of target bacterial groups. **(A)** Transcript expression of *ApBD1* and *ApLBP/BPI1* in scallop hemocytes injected with *Ap*BD1-dsRNA (red bar) and *Ap*LBP/BPI1dsRNA (blue bar) compared to GFP-dsRNA (gray bar), considered as control of specific gene silencing. Values are represented as the mean ± SE, considering the gene expression in GFP-dsRNA-injected animals as 100%. **(B)** Relative abundances of target bacterial groups present in the hemolymph of dsRNA injected scallops determined by qPCR and absolute quantification. **(C)** Number of total copies of specific bacterial 16S rDNA gene for *Vibrio* spp. present in dsRNA injected scallops. Values are represented as the mean ± SE and asterisks indicate significant differences compared with GFP-dsRNA-injected scallops (*P < 0.05, ***P < 0.005). **(D)** cultivable *Vibrio* spp. present in hemolymph from dsRNA injected scallops. Values are represented as the mean number of colony forming units/ml of hemolymph ± SE.

Since we detected significant shifts of bacterial groups in scallop hemolymph together with the overexpression of antimicrobial effectors, we focused on the role of *ApBD1* and *ApLBP/BPI1* in shaping the scallop microbiota. Thus, we performed a RNA interference silencing approach by injecting sequence-specific dsRNA in non-immune stimulated scallops. Subsequently, we quantified the 16S rDNA of target bacterial groups present in scallop hemolymph by qPCR (**Figure 5**). Results showed that transcript expressions of *ApBD1* and *ApLBP/BPI1* in hemocytes from scallops injected with *Ap*BD1-dsRNA and *Ap*LBP/BPI1-dsRNA were significant suppressed by ~94% and ~45%, respectively, compared to scallops injected with GFP-dsRNA as control after 48 h (**Figure 5A**). In addition, the expressions of *ApBD1* and *ApLBP/BPI1* in scallops injected with GFP-dsRNA were consistent with the basal expression levels observed in hemocytes from non-stimulated scallops (**Figure S6**). Then, we assessed the relative abundances of Gamma-, Epsilon-, and Betaproteobacteria, and Firmicutes from every experimental group. Particularly, Gammaproteobacteria significantly increased when the expression of antimicrobial effectors was suppressed compared to the control group (**Figure 5B**). We observed the highest relative abundance of Gammaproteobacteria in *Ap*LBP/BPI1-dsRNA injected scallops (81.8%) followed by *Ap*BD1-dsRNA (69.3%) and GFP-dsRNA (35.9%) injected scallops. Concomitantly, the increase in Gammaproteobacteria was associated with a significant decrease in undetermined bacterial groups (named as others). Specifically, *Ap*LBP/BPI1-dsRNA injected scallops displayed the lowest abundance of undetermined groups (9.3%) followed by *Ap*BD1-dsRNA (18.4%) and GFP-dsRNA (35.9%) injected scallops. Epsilonproteobacteria also displayed a significant decrease in *Ap*LBP/BPI1-dsRNA injected (5.7%) compared to control scallops (28.7%). Firmicutes and Betaproteobacteria did not show any significant shift in their relative abundances among experimental conditions. Moreover, relative abundances of bacterial groups from scallops injected with GFP-dsRNA were non-significantly different from SW-injected scallops.

In parallel, we detected a significant increase of the number of total copies of specific bacterial 16S rDNA gene from the genus *Vibrio* in scallops silenced for *ApBD1* or *ApLBP/BPI1* (**Figure 5C**). In terms of the relative abundance, *Vibrio* spp. corresponded to 29% and 22% of total bacteria in *Ap*LBP/BPI1- and *Ap*BD1-dsRNA injected scallops, respectively, while *Vibrio* spp. only represented 2% of total bacteria in GFP-dsRNA injected scallops. Finally, to confirm that the increase in *Vibrio* was due to cultivable *Vibrio* spp., hemolymph from all dsRNA-injected scallops were plated onto TCBS selective media. Results showed a significant increased number of *Vibrio* spp. c.f.u. in *Ap*LBP/BPI1- and *Ap*BD1-dsRNA injected scallops compared to the GFP-control group (*P*<0.05) (**Figure 5D**).

## Discussion

Results obtained in the present study shows that relative abundances of different bacterial groups found in the scallop hemolymph could rapidly change during the immune response. We also establish that the abundances of major shifted bacterial groups were restored within a period of 7 days after immune challenge in a controlled environment. Importantly, the silencing of expression of the two antimicrobial effectors in non-immune stimulated scallops demonstrate that shifts of some major bacterial groups’ abundances are related to the expression of antimicrobial effectors. Indeed, silenced scallops for both, *ApBD1* and *ApLBP/BPI1* antimicrobials exhibited proliferation of *Vibrio* spp. in their hemolymph. Overall, these results depict the delicate balance that exists between the immune response of *A. purpuratus* and hemolymph microbiota, indicating that host antimicrobial peptides and proteins could modulate the abundances of certain groups of bacteria such as *Vibrio*.

The analysis of hemolymph bacterial composition from non-stimulated scallops at the individual level reveal that scallops display a variable bacterial community, with both common and distinct bacterial groups among individuals. Considering the common phyla, scallop hemolymph is dominated by Proteobacteria, Spirochaetes, Bacteroidetes, Firmicutes and Gracilibacteria. Some of these phyla have been described also as predominant bacterial groups in other marine invertebrates. For example, Proteobacteria and/or Bacteroidetes are dominant phyla in the hemolymph of the mussel *Mytilus coruscus* (47), the oyster *Crassostrea gigas* (48), certain crustaceans (49), and in gonads from the scallop *Pecten maximus* (50). Yet, high variability in bacterial community composition and the lack of a common bacterial core between all scallop individuals is more evident at the class and genera level. Considering that scallops were acclimatized for two weeks in controlled conditions prior experiments, the variability on bacterial communities found between individuals is unlikely associated to the marine environment, as proposed in a previous study which characterized the microbiota from scallops sampled from the field (34). One single individual displays a microbiota composition quite different from the observed for the other individuals, which suggests the existence of higher microbiota variability in non-stimulated scallops. In oysters, the stability of the hemolymph microbiota and the assembly of the microbial community has been related to the host genotype (51), suggesting that the hemolymph microbiome is not a simple reflection of bivalve filtering lifestyle (48). Consequently, both extrinsic (environmental) and intrinsic (host) factors are at play in shaping and mediating the bacterial communities of aquatic organisms (52). As well, bacterial community variability has been related to different levels of basal expression of certain antimicrobial effectors among individuals (29), so each animal would represent bacterial niches with unique characteristics (30, 53). Indeed, consistent differences have been reported between host microbiota and environmental bacterial communities in a large number of marine invertebrates. These evidences suggest the existence of mechanisms for selection, adaptation, and regulation between hosts and associated microorganisms (30, 54–57).

The composition of the hemolymph microbiota significantly changed at 48 h post immune challenge, a result previously observed from whole scallops directly sampled from the field (34). The evidence that scallop microbiota shifts during the immune response at both, controlled conditions and natural environment, suggests a strong host genetic effect in this process, as previously demonstrated in *Hydra* (56). In the present study, we found that (i) significant shifts in bacterial groups can be detected as early as 24 h after immune challenge and (ii) shifted abundances of most bacterial groups are reestablished within 7 days post immunostimulation. These results give new insights into the kinetics of the scallop microbiota modulation, showing that abundances of some bacterial populations can be rapidly shifted and restored in the scallop after the immune response. In the oyster *C. gigas*, recent studies have shown that hemolymph microbiota dynamics are subject to internal microbiome forces and host-related factors, such as genetics, that contribute to long-term stability (48, 58). Indeed, no significant bacterial shifts were detected in scallop hemolymph at phylum level, and the significant changes observed at class level during immunostimulation were reestablished within 7 days, supporting the idea of stability in the dynamics of the marine bivalve hemolymph microbiome (48, 58).

The significant increase in the Gammaproteobacteria class, in which *Vibrio* only corresponds to a minor fraction of total Gammaproteobacteria abundance, together with the significant decrease of Epsilonproteobacteria and Deltaproteobacteria, is considered as a clear sign of microbiota destabilization in immunostimulated scallops. Studies from other marine bivalves have proposed that processes such as infections, temperature stress, contaminants, among others, can trigger the destabilization of the microbiota, “opening the door” to opportunistic pathogens (32, 48, 59–61). In the present work, we specifically induce the immune activation with a heat-killed *Vibrio* to exclude the pathogen-microbe interaction that could occur during a real pathogenic infection. The selection of the four antimicrobial effectors to confirm scallop immune activation was based on their involvement in the immune response and on their antibacterial properties described in this and others bivalves species (12, 15, 62–64). Thus, bacterial shifts observed were possibly shaped in part by the host response and consequently, the silencing of antimicrobial effectors expressed by the host was a functional approach to validate this premise.

*Ap*BD1 and *Ap*LBP/BPI1 effectors were previously characterized as *A. purpuratus* antimicrobial effector genes (12, 15). Both genes exhibited high levels of expression during bacterial challenge and were expressed at basal level by hemocytes and other tissues. Furthermore, *Ap*BD1 was recently shown to entrap *Staphylococcus aureus* in peptide aggregates similar to those reported to the oyster Big defensin *Cg*-BigDef1 (63). Although *Ap*LBP/BPI1 has not been functional characterized yet, its oyster homologue *Cg*BPI display strong antibacterial activity by membrane permeabilization of gram-negative bacteria (62). The transcript silencing of these genes in non-stimulated scallops indicate that both antimicrobials are involved in the regulation of *A. purpuratus* bacterial microbiota. Interestingly, consistent changes in Gamma- and Epsilonproteobacteria abundances were detected when these effectors were overexpressed or silenced. This result suggests that changes in bacterial groups abundances might be occurring at lower taxonomic levels within those bacterial classes. Certainly, considering the antibacterial activities reported for these effectors, *Ap*BD1 and *Ap*LBP/BPI1 could specifically regulate certain target bacterial groups, controlling their abundance in the hemolymph. Also, antimicrobials could be acting indirectly through the modulation of certain bacterial groups that interact with the bacterial groups targeted in this study, for instance by antagonistic interactions (65). In parallel, diverse cellular reactions and humoral effectors are produced during the scallop immune response. Thus, additional immune processes might be associated with bacterial shifts regulation, such as expression of further antimicrobial effectors, immunomodulators, and cellular responses (22, 30, 34, 55).

Overall, our results bring out the importance of the scallop humoral immune response not only in defense against external pathogens, but also in regulating the proliferation and maintaining specific levels of certain bacterial groups. In other invertebrate species such as *Marsupenaeus japonicus* or *Drosophila melanogaster*, silencing or knockdown of antimicrobial peptides can cause host death, due to an exacerbated proliferation of bacteria in hemolymph or tissues (22, 29). Antimicrobial peptides expressed in *Hydra* select specific bacterial colonization, changing the structure of the bacterial community when their expression is altered (66). In this study, we depicted the kinetics of microbiota modification during the scallop immune response. We also defined the functional interaction between the expression of two antimicrobial effectors and the hemolymph microbiota of *A. purpuratus*. Future research on the beneficial or detrimental effect of bacterial composition shifts in the immune capacity of scallops will allow us to contribute with the design of management aquaculture strategies. Also, which antimicrobial peptides and proteins specifically shaped target bacterial groups from scallop microbiota will be a pertinent information to implement future genetic breading programs in *A. purpuratus*.

## Data Availability Statement

The datasets presented in this study can be found in online repositories. The names of the repository/repositories and accession number(s) can be found below: https://www.ncbi.nlm.nih.gov/genbank/, PRJNA639911.

## Author Contributions

RG, RDR, KB, and PS contributed to the conception and design of the study. RG, AG, and RR contributed to the acquisition of data. RG and AG created the databases and performed the statistical analysis. RG and PS worked on analysis, interpretation of data, and wrote the first draft of the manuscript. All authors contributed to the article and approved the submitted version.

## Funding

This study was supported by the following research funding programs: FONDECYT No 11150009 to PS. RG. was supported by the PhD student fellowship CONICYT-PFCHA/DOCTORADO NACIONAL/2016 – 21160980. RDR was supported by the Brazilian funding agency CNPq (Grant Numbers 406530/2016-5 and 307032/2018-3).

## Conflict of Interest

The authors declare that the research was conducted in the absence of any commercial or financial relationships that could be construed as a potential conflict of interest.

## References

[1] Wang S Zhang J Jiao W Li J Xun X Sun Y Scallop genome provides insights into evolution of bilaterian karyotype and development. Nat Ecol Evol (2017) 1(5):120. 10.1038/s41559-017-0120 28812685 PMC10970998

[2] Gram L Melchiorsen J Bruhn JB Antibacterial activity of marine culturable bacteria collected from a global sampling of ocean surface waters and surface swabs of marine organisms. Mar Biotechnol (NY) (2010) 12(4):439–51. 10.1007/s10126-009-9233-y 19823914

[3] Inglis SD Kristmundsson A Freeman MA Levesque M Stokesbury K Gray meat in the Atlantic sea scallop, *Placopecten magellanicus*, and the identification of a known pathogenic scallop apicomplexan. J Invertebr Pathol (2016) 141:66–75. 10.1016/j.jip.2016.10.008 27810289

[4] Xing J Lin T Zhan W Variations of enzyme activities in the haemocytes of scallop *Chlamys farreri* after infection with the acute virus necrobiotic virus (AVNV). Fish Shellfish Immunol (2008) 25(6):847–52. 10.1016/j.fsi.2008.09.008 18930154

[5] Liu R Qiu L Yu Z Zi J Yue F Wang L Identification and characterisation of pathogenic *Vibrio splendidus* from Yesso scallop (*Patinopecten yessoensis*) cultured in a low temperature environment. J Invertebr Pathol (2013) 114(2):144–50. 10.1016/j.jip.2013.07.005 23911357

[6] González R Muñoz K Brokordt K Schmitt P Scallop Immunology. In: Reference Module in Life Sciences. Massachusetts: Elsevier (2019). 10.1016/B978-0-12-809633-8.20896-0

[7] Escoubas J-M Gourbal B Duval D Green TJ Charrière GM Destoumieux-Garzón D Immunity in Molluscs. In: Ratcliffe MJH, editor. Encyclopedia of Immunobiology. Oxford: Academic Press (2016). 10.1016/B978-0-12-374279-7.12004-1

[8] Gerdol M De Moro G Manfrin C Venier P Pallavicini A Big defensins and mytimacins, new AMP families of the Mediterranean mussel *Mytilus galloprovincialis*. Dev Comp Immunol (2012) 36(2):390–9. 10.1016/j.dci.2011.08.003 21871485

[9] Allam B Espinosa EP Bivalve immunity and response to infections: Are we looking at the right place? Fish Shellfish Immunol (2016) 53:4–12. 10.1016/j.fsi.2016.03.037 27004953

[10] Yang J Luo J Zheng H Lu Y Zhang H Cloning of a big defensin gene and its response to *Vibrio parahaemolyticus* challenge in the noble scallop *Chlamys nobilis* (Bivalve: Pectinidae). Fish Shellfish Immunol (2016) 56:445–449. 10.1016/j.fsi.2016.07.030 27474446

[11] Zhao J Song L Li C Ni D Wu L Zhu L Molecular cloning, expression of a big defensin gene from bay scallop *Argopecten irradians* and the antimicrobial activity of its recombinant protein. Mol Immunol (2007) 44(4):360–8. 10.1016/j.molimm.2006.02.025 16597463

[12] Gonzalez R Brokordt K Carcamo CB Coba de la Pena T Oyanedel D Mercado L Molecular characterization and protein localization of the antimicrobial peptide big defensin from the scallop *Argopecten purpuratus* after *Vibrio splendidus* challenge. Fish Shellfish Immunol (2017) 68:173–9. 10.1016/j.fsi.2017.07.010 28690141

[13] Zhao J Song L Li C Zou H Ni D Wang W Molecular cloning of an invertebrate goose-type lysozyme gene from *Chlamys farreri*, and lytic activity of the recombinant protein. Mol Immunol (2007) 44(6):1198–208. 10.1016/j.molimm.2006.06.008 16911829

[14] He C Yu H Liu W Su H Shan Z Bao X A goose-type lysozyme gene in Japanese scallop (*Mizuhopecten yessoensis*): cDNA cloning, mRNA expression and prom oter sequence analysis. Comp Biochem Physiol B Biochem Mol Biol (2012) 162(1-3):34–43. 10.1016/j.cbpb.2012.02.002 22366552

[15] González R Brokordt K Rojas R Schmitt P Molecular characterization and expression patterns of two LPS binding /bactericidal permeability-increasing proteins (LBP/BPIs) from the scallop *Argopecten purpuratus*. Fish Shellfish Immunol (2020) 97:12–7. 10.1016/j.fsi.2019.12.032 31843699

[16] Li C Song L Zhao J Zhu L Zou H Zhang H Preliminary study on a potential antibacterial peptide derived from histone H2A in hemocytes of scallop *Chlamys farreri*. Fish Shellfish Immunol (2007) 22(6):663–72. 10.1016/j.fsi.2006.08.013 17049445

[17] Jiang W Lin F Fang J Gao Y Du M Fang J Transcriptome analysis of the Yesso scallop, *Patinopecten yessoensis* gills in response to water temperature fluctuations. Fish Shellfish Immunol (2018) 80:133–40. 10.1016/j.fsi.2018.05.038 29860069

[18] Pauletto M Milan M Moreira R Novoa B Figueras A Babbucci M Deep transcriptome sequencing of *Pecten maximus* hemocytes: a genomic resource for bivalve immunology. Fish Shellfish Immunol (2014) 37(1):154–65. 10.1016/j.fsi.2014.01.017 24486903

[19] Ventoso P Pazos AJ Pérez-Parallé ML Blanco J Triviño JC Sánchez JL RNA-Seq Transcriptome Profiling of the Queen Scallop (*Aequipecten opercularis*) Digestive Gland after Exposure to Domoic Acid-Producing Pseudo-nitzschia. Toxins (2019) 11(2):97. 10.3390/toxins11020097 PMC6410316 30736356

[20] Flores-Herrera P Farlora R Gonzalez R Brokordt K Schmitt P De novo assembly, characterization of tissue-specific transcriptomes and identification of immune related genes from the scallop *Argopecten purpuratus*. Fish Shellfish Immunol (2019) 89:505–15. 10.1016/j.fsi.2019.03.069 30940577

[21] Hooper LV Littman DR Macpherson AJ Interactions between the microbiota and the immune system. Science (2012) 336(6086):1268–73. 10.1126/science.1223490 PMC4420145 22674334

[22] Ryu JH Kim SH Lee HY Bai JY Nam YD Bae JW Innate immune homeostasis by the homeobox gene caudal and commensal-gut mutualism in Drosophila. Science (2008) 319(5864):777–82. 10.1126/science.1149357 18218863

[23] Zhao Q Elson CO Adaptive immune education by gut microbiota antigens. Immunology (2018) 154(1):28–37. 10.1111/imm.12896 29338074 PMC5904715

[24] Abt MC Osborne LC Monticelli LA Doering TA Alenghat T Sonnenberg GF Commensal bacteria calibrate the activation threshold of innate antiviral immunity. Immunity (2012) 37(1):158–70. 10.1016/j.immuni.2012.04.011 PMC3679670 22705104

[25] Valdes AM Walter J Segal E Spector TD Role of the gut microbiota in nutrition and health. BMJ (2018) 361:k2179. 10.1136/bmj.k2179 29899036 PMC6000740

[26] Salzman NH Paneth cell defensins and the regulation of the microbiome: detente at mucosal surfaces. Gut Microbes (2010) 1(6):401–6. 10.4161/gmic.1.6.14076 PMC3056107 21468224

[27] Sassone-Corsi M Raffatellu M No vacancy: how beneficial microbes cooperate with immunity to provide colonization resistance to pathogens. J Immunol (2015) 194(9):4081–7. 10.4049/jimmunol.1403169 PMC4402713 25888704

[28] You JS Yong JH Kim GH Moon S Nam KT Ryu JH Commensal-derived metabolites govern *Vibrio cholerae* pathogenesis in host intestine. Microbiome (2019) 7(1):132. 10.1186/s40168-019-0746-y 31521198 PMC6744661

[29] Wang XW Xu JD Zhao XF Vasta GR Wang JX A shrimp C-type lectin inhibits proliferation of the hemolymph microbiota by maintaining the expression of antimicrobial peptides. J Biol Chem (2014) 289(17):11779–90. 10.1074/jbc.M114.552307 PMC4002086 24619414

[30] Franzenburg S Walter J Künzel S Wang J Baines JF Bosch TC Distinct antimicrobial peptide expression determines host species-specific bacterial associations. Proc Natl Acad Sci U S A (2013) 110(39):E3730–8. 10.1073/pnas.1304960110 PMC3785777 24003149

[31] Wang GL Xia XL Li XL Dong SJ Li JL Molecular characterization and expression patterns of the big defensin gene in freshwater mussel (*Hyriopsis cumingii*). Genet Mol Res (2014) 13(1):704–15. 10.4238/2014.January.29.1 24615035

[32] Clerissi C de Lorgeril J Petton B Lucasson A Escoubas JM Gueguen Y Microbiota Composition and Evenness Predict Survival Rate of Oysters Confronted to Pacific Oyster Mortality Syndrome. Front Microbiol (2020) 11:311. 10.3389/fmicb.2020.00311 32174904 PMC7056673

[33] de Lorgeril J Lucasson A Petton B Toulza E Montagnani C Clerissi C Immune-suppression by OsHV-1 viral infection causes fatal bacteraemia in Pacific oysters. Nat Commun (2018) 9(1):4215. 10.1038/s41467-018-06659-3 30310074 PMC6182001

[34] Muñoz K Flores-Herrera P Gonçalves AT Rojas C Yáñez C Mercado L The immune response of the scallop *Argopecten purpuratus* is associated with changes in the host microbiota structure and diversity. Fish Shellfish Immunol (2019) 91:241–50. 10.1016/j.fsi.2019.05.028 31100440

[35] L. Committee on Acute Exposure Guideline T. Committee on T. Board on Environmental Studies and S. Division on Earth and Life and C. National Research Acute Exposure Guideline Levels for Selected Airborne Chemicals: Volume 16. Washington (DC: National Academies Press (US) Copyright 2014 by the National Academy of Sciences (2014). All rights reserved. 10.17226/18707

[36] Rojas R Miranda CD Opazo R Romero J Characterization and pathogenicity of *Vibrio splendidus* strains associated with massive mortalities of commercial hatchery-reared larvae of scallop *Argopecten purpuratus* (Lamarck, 1819). J Invertebr Pathol (2015) 124:61–9. 10.1016/j.jip.2014.10.009 25450196

[37] Pfaffl MW A new mathematical model for relative quantification in real-time RT-PCR. Nucleic Acids Res (2001) 29(9):e45–5. 10.1093/nar/29.9.e45 PMC55695 11328886

[38] Caporaso JG Kuczynski J Stombaugh J Bittinger K Bushman FD Costello EK QIIME allows analysis of high-throughput community sequencing data. Nat Methods (2010) 7(5):335–6. 10.1038/nmeth.f.303 PMC3156573 20383131

[39] Bolyen E Rideout JR Dillon MR Bokulich NA Abnet CC Al-Ghalith GA Reproducible, interactive, scalable and extensible microbiome data science using QIIME 2. Nat Biotechnol (2019) 37(8):852–7. 10.1038/s41587-019-0209-9 PMC7015180 31341288

[40] Callahan BJ McMurdie PJ Rosen MJ Han AW Johnson AJA Holmes SP DADA2: High-resolution sample inference from Illumina amplicon data. Nat Methods (2016) 13(7):581–3. 10.1038/nmeth.3869 PMC4927377 27214047

[41] Callahan BJ McMurdie PJ Holmes SP Exact sequence variants should replace operational taxonomic units in marker-gene data analysis. Isme J (2017) 11(12):2639–43. 10.1038/ismej.2017.119 PMC5702726 28731476

[42] McDonald D Price MN Goodrich J Nawrocki EP DeSantis TZ Probst A An improved Greengenes taxonomy with explicit ranks for ecological and evolutionary analyses of bacteria and archaea. Isme J (2012) 6(3):610–8. 10.1038/ismej.2011.139 PMC3280142 22134646

[43] Edgar RC Updating the 97% identity threshold for 16S ribosomal RNA OTUs. Bioinformatics (2018) 34(14):2371–5. 10.1093/bioinformatics/bty113 29506021

[44] Lozupone C Knight R UniFrac: a new phylogenetic method for comparing microbial communities. Appl Environ Microbiol (2005) 71(12):8228–35. 10.1128/AEM.71.12.8228-8235.2005 PMC1317376 16332807

[45] Lozupone CA Hamady M Kelley ST Knigh R Quantitative and qualitative beta diversity measures lead to different insights into factors that structure microbial communities. Appl Environ Microbiol (2007) 73(5):1576–85. 10.1128/aem.01996-06 PMC1828774 17220268

[46] Parks DH Tyson GW Hugenholtz P Beiko RG STAMP: statistical analysis of taxonomic and functional profiles. Bioinformatics (2014) 30(21):3123–4. 10.1093/bioinformatics/btu494 PMC4609014 25061070

[47] Li YF Chen YW Xu JK Ding WY Shao AQ Zhu YT Temperature elevation and *Vibrio cyclitrophicus* infection reduce the diversity of haemolymph microbiome of the mussel *Mytilus coruscus*. Sci Rep (2019) 9(1):16391. 10.1038/s41598-019-52752-y 31704981 PMC6841970

[48] Lokmer A Mathias Wegner K Hemolymph microbiome of Pacific oysters in response to temperature, temperature stress and infection. Isme J (2015) 9(3):670–82. 10.1038/ismej.2014.160 PMC4331581 25180968

[49] Ooi MC Goulden EF Smith GG Bridle AR Haemolymph microbiome of the cultured spiny lobster *Panulirus ornatus* at different temperatures. Sci Rep (2019) 9(1):1677. 10.1038/s41598-019-39149-7 30737466 PMC6368590

[50] Lasa A Mira A Camelo-Castillo A Belda-Ferre P Romalde JL Characterization of the microbiota associated to *Pecten maximus* gonads using 454-pyrosequencing. Int Microbiol (2016) 19(2):93–9. 10.2436/20.1501.01.267 27845496

[51] Wegner KM Volkenborn N Peter H Eiler A Disturbance induced decoupling between host genetics and composition of the associated microbiome. BMC Microbiol (2013) 13:252. 10.1186/1471-2180-13-252 24206899 PMC3840651

[52] Melissa LP Ward JE Microbial Ecology of the Bivalvia, with an Emphasis on the Family Ostreidae. J Shellfish Res (2018) 37(4):793–806. 10.2983/035.037.0410

[53] Wang XW Wang JX Crustacean hemolymph microbiota: Endemic, tightly controlled, and utilization expectable. Mol Immunol (2015) 68(2 Pt B):404–11. 10.1016/j.molimm.2015.06.018 26153452

[54] Augustin R Schröder K Murillo Rincón AP Fraune S Anton-Erxleben F Herbst E-M A secreted antibacterial neuropeptide shapes the microbiome of Hydra. Nat Commun (2017) 8(1):698. 10.1038/s41467-017-00625-1 28951596 PMC5614986

[55] Bevins CL Salzman NH The potter’s wheel: the host’s role in sculpting its microbiota. Cell Mol Life Sci (2011) 68(22):3675–85. 10.1007/s00018-011-0830-3 PMC3222938 21968920

[56] Fraune S Bosch TCG Long-term maintenance of species-specific bacterial microbiota in the basal metazoan Hydra. Proc Natl Acad Sci U S A (2007) 104(32):13146–51. 10.1073/pnas.0703375104 PMC1934924 17664430

[57] Cullen TW Schofield WB Barry NA Putnam EE Rundell EA Trent MS Gut microbiota. Antimicrobial peptide resistance mediates resilience of prominent gut commensals during inflammation. Science (2015) 347(6218):170–5. 10.1126/science.1260580 PMC4388331 25574022

[58] Lokmer A Goedknegt MA Thieltges DW Fiorentino D Kuenzel S Baines JF Spatial and Temporal Dynamics of Pacific Oyster Hemolymph Microbiota across Multiple Scales. Front Microbiol (2016) 7:1367. 10.3389/fmicb.2016.01367 27630625 PMC5006416

[59] Egan S Gardiner M Microbial Dysbiosis: Rethinking Disease in Marine Ecosystems. Front Microbiol (2016) 7:991. 10.3389/fmicb.2016.00991 27446031 PMC4914501

[60] Bachere E Rosa RD Schmitt P Poirier AC Merou N Charriere GM The new insights into the oyster antimicrobial defense: Cellular, molecular and genetic view. Fish Shellfish Immunol (2015) 46(1):50–64. 10.1016/j.fsi.2015.02.040 25753917

[61] Destoumieux-Garzón D Canesi L Oyanedel D Travers M-A Charrière GM Pruzzo C Vibrio–bivalve interactions in health and disease. Environ Microbiol (2020) 4323–41. 10.1111/1462-2920.15055 32363732

[62] Gonzalez M Gueguen Y Destoumieux-Garzon D Romestand B Fievet J Pugniere M Evidence of a bactericidal permeability increasing protein in an invertebrate, the *Crassostrea gigas* Cg-BPI. Proc Natl Acad Sci U S A (2007) 104(45):17759–64. 10.1073/pnas.0702281104 PMC2077063 17965238

[63] Stambuk F Ojeda C Schmitt P Big Defensin BD1 from the scallop *Argopecten purpuratus* is an antimicrobial peptide which entraps bacteria through nanonets formation. bioRxiv (2020). 10.1101/2020.02.25.965327 34710565

[64] Zhao J Song L Li C Zou H Ni D Wang W Molecular cloning of an invertebrate goose-type lysozyme gene from *Chlamys farreri*, and lytic activity of the recombinant protein. Mol Immunol (2007) 44(6):1198–208. 10.1016/j.molimm.2006.06.008 16911829

[65] García-Bayona L Comstock LE Bacterial antagonism in host-associated microbial communities. Science (2018) 361(6408):eaat2456. 10.1126/science.aat2456 30237322

[66] Fraune S Augustin R Anton-Erxleben F Wittlieb J Gelhaus C Klimovich VB In an early branching metazoan, bacterial colonization of the embryo is controlled by maternal antimicrobial peptides. Proc Natl Acad Sci U S A (2010) 107(42):18067–72. 10.1073/pnas.1008573107 PMC2964230 20921390

