# Supplementary material for: Host Defense Effectors Expressed by Hemocytes Shape the Bacterial Microbiota From the Scallop Hemolymph
Source: Front Immunol. 2020 Nov 12;11:599625. doi: 10.3389/fimmu.2020.599625 (PMC7689009; doi:10.3389/fimmu.2020.599625)
Supplement: Supplementary file 1 [file DataSheet_1.docx]

Supplementary Material


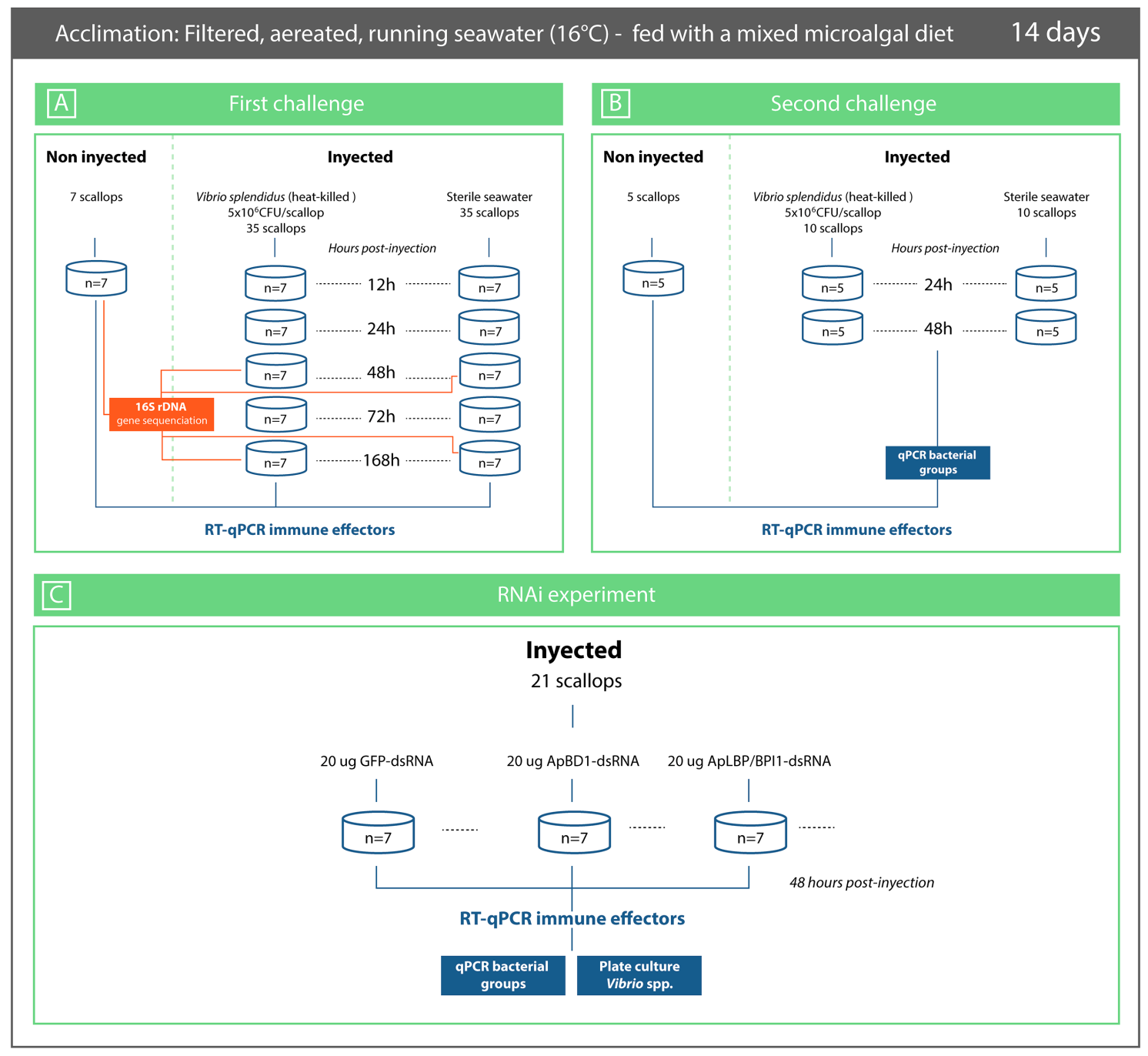


**Supplementary Figure 1.** Schematic representation of 3 experiments performed in the present study. **A.** First immune challenge. **B.** Second immune challenge. **C.** RNA interference experiment using non-immunostimulated scallops. Sampling time points, number of scallop individuals sampled at each time point and analysis performed from each sample are indicated in the three independent experimental challenges.





**Supplementary Figure 2.** Rarefaction curves obtained from total number of OTUs obtained from each samples analyzed by 16S rDNA deep amplicon sequencing.





**Supplementary Figure 3.** **Alpha diversity indices (Shannon and Simpson) and richness indices Chao1 y Faith PD (Faith's Phylogenetic Diversity) found in hemolymph bacterial microbiota from analyzed scallops.** VS: Vibrio-injected scallops; SW: seawater-injected scallops. Injected scallops were sampled at 48 and 168 h post injection.


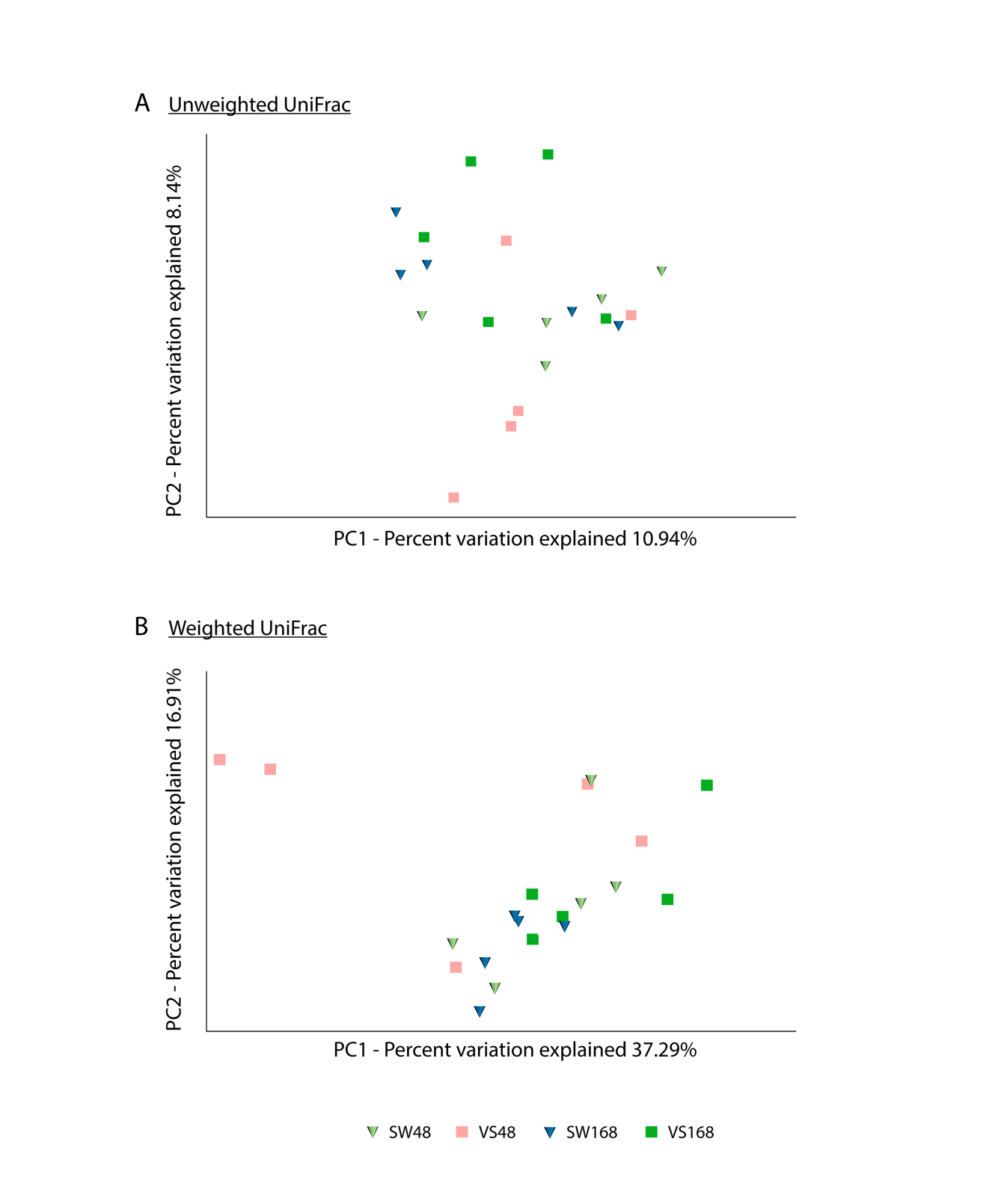


**Supplementary Figure 4.** **Beta diversity analysis of the scallop hemolymph microbiota by Principal Coordinate Analysis (PCoA).** Unweighted (**A**) and Weighted of Unifrac (**B**) distances were calculated for hemolymph bacterial microbiota. VS: Vibrio-injected scallops; SW: seawater-injected scallops. Injected scallops were sampled at 48 and 168 h post injection.





**Supplementary Figure 5.** **Basal expression of *ApBD1, ApLBP/BPI1, ApLBP/BPI2* and *ApGlys* immune response genes in hemocytes of unstimulated scallops.** The data presented correspond to the difference between the C_q_ value of target gene and the reference gene (β actin). Data are expressed as mean ± ES.





**Supplementary Figure 6.** **Validation of overexpression of antimicrobial effectors in the second immune challenge performed in the present study.** Data correspond to the relative expression of *ApBD1* and *ApLBP/BPI1* in hemocytes of *Vibrio*-injected scallops (VS) and seawater-injected scallops (SW) after 24 and 48 h post challenge. SW-injected scallops were considered as injury control condition. Relative expression was calculated using non-stimulated scallops as control group, where gene expression values were considered 1. Graphed data are represented as the mean ± ES (n = 5). Asterisks indicate significant differences compared to SW-injected scallops (*P* <0.05).
