# Supplementary material for: Host Defense Effectors Expressed by Hemocytes Shape the Bacterial Microbiota From the Scallop Hemolymph
Source: Front Immunol. 2020 Nov 12;11:599625. doi: 10.3389/fimmu.2020.599625 (PMC7689009; doi:10.3389/fimmu.2020.599625)
Supplement: Supplementary file 2 [file Table_1.docx]

Supplementary Material

| **Supplementary Table 1** | |  | | |  | | |  |
| --- | --- | --- | --- | --- | --- | --- | --- | --- |
| **Nucleotide sequence of primers used in this study** | | | |  | |  |  |  |
|  | **Primer name** | | **Sequence (5´-3´)** | **Source or reference** | |  |  |  |
| **RT-qPCR Inmune effectors** |  | |  |  | |  |  |  |
| *ApLBP/BPI2* | qApLBP/BPI2-F | | CTGCTGCCAACCGTTCTGC | (1) | |  |  |  |
|  | qApLBP/BPI2-R | | CGCATGTGCAGATCAACCTGG |  | |  |  |  |
| *ApLBP/BPI1* | qApLBP/BPI-F | | GTGAAGGCACCAAATGCAACCG | (1) | |  |  |  |
|  | qApLBP/BPI-R | | GGAGCGAAGATAACCGTCAGAGT |  | | |  |  |
| *qApBD1* | qApBD1-F | | TGGCAACAGCGGATGGTGT | (2) | |  |  |  |
|  | qApBD1-R | | AACGCCTAAGTTCCCACCTCG |  | |  |  |  |
| *qApGlyz* | qApGlyz-F | | GGAGACCATCACCATGCTTACG | This study | |  |  |  |
|  | qApGlyz-R | | TGGGAAATATGTGCGCAGCTGTC |  | |  |  |  |
| *qApβ-actin* | qApβ-actin-F | | CACTGCTCTTGCTCCACAAAC | (3) | |  |  |  |
|  | qApβ-actin-R | | GAAGGTGGACAGAGATGCCAA |  | |  |  |  |
| **qPCR bacterial groups** |  | |  |  | |  |  |  |
| Eubacteria | 926F | | AAACTCAAAKGAATTGACGG | (4) | |  |  |  |
|  | 1062R | | CTCACRRCACGAGCTGAC |  | |  |  |  |
| Firmicutes | Firm934F | | GGAGYATGTGGTTTAATTCGAAGCA | (5) | |  |  |  |
|  | Firm1060R | | AGCTGACGACAACCATGCAC |  | |  |  |  |
| Epsilonproteobacteria | Epsilon940F | | TAGGCTTGACATTGATAGAATC | (6) | |  |  |  |
|  | Epsilon1129R | | CTTACGAAGGCAGTCTCCTTA |  | |  |  |  |
| Gammaproteobacteria | 1080ƴF | | TCGTCAGCTCGTGTYGTGA | (4) | |  |  |  |
|  | ƴ1202R | | CGTAAGGGCCATGATG |  | |  |  |  |
| Betaproteobacteria | Beta979F | | AACGCGAAAAACCTTACCTACC | (6) | |  |  |  |
|  | Beta1130R | | TGCCCTTTCGTAGCAACTAGTG |  | |  |  |  |
| Vibrio spp. | 567F | | GGCGTAAAGCGCATGCAGGT | (7) | |  |  |  |
|  | 680R | | GAAATTCTACCCCCCTCTACAG |  | |  |  |  |
| **PCR Eubacteria** |  | |  |  | |  |  |  |
| Bacterial 16S rRNA | 27f-CM | | AGAGTTTGATCMTGGCTCAG | (8) | |  |  |  |
|  | S-D-Bact-1392-a-A-19 | | TGACGGGCGGTGTGTACAA | (9) | |  |  |  |
| **Primers for RNAi** |  | |  |  | |  |  |  |
| *dsApLBP/BPI1* | dsApLBP/BPI1-F | | **T7**-GACTATGGACTACAAGAACC | This study | |  |  |  |
|  | dsApLBP/BPI1-R | | **T7**-CCATGTTCGTGTCCATAGTG |  | |  |  |  |
| *dsApBD1* | dsApBD1-F | | **T7-**TGTAATGGCCATCGTGTGTC | This study | |  |  |  |
|  | dsApBD1-R | | **T7**-TCTCTCGTATGAGCGGCATG |  | |  |  |  |
| *dsGFP* | dsGFP-F | | **T7**-GAGCAAGGGCGAGGAGCTGT | (3) | |  |  |  |
|  | dsGFP-R | | **T7**-CCTCCTTGAAGTCGATGCCC |  | |  |  |  |
| **T7-tag sequence**: TAATACGACTCACTATAGG. | | | |  | |  |  |  |

**References**

1. R. González, K. Brokordt, R. Rojas and P. Schmitt. Molecular characterization and expression patterns of two LPS binding /bactericidal permeability-increasing proteins (LBP/BPIs) from the scallop *Argopecten purpuratus*. *Fish Shellfish Immunol*. (2020), 97, 12-17. doi:<https://doi.org/10.1016/j.fsi.2019.12.032>

2. D. Oyanedel, R. Gonzalez, K. Brokordt, P. Schmitt and L. Mercado: Insight into the messenger role of reactive oxygen intermediates in immunostimulated hemocytes from the scallop *Argopecten purpuratus*. *Dev Comp Immunol.* (2016), 65, 226-230. doi:10.1016/j.dci.2016.07.015

3. D. Oyanedel, R. Gonzalez, P. Flores-Herrera, K. Brokordt, R. D. Rosa, L. Mercado et al. Molecular characterization of an inhibitor of NF-kappa B in the scallop *Argopecten purpuratus*: First insights into its role on antimicrobial peptide regulation in a mollusk. *Fish Shellfish Immunol.*(2016) 52, 85-93. doi:10.1016/j.fsi.2016.03.021

4. T. Bacchetti De Gregoris, N. Aldred, A. S. Clare and J. G. Burgess. Improvement of phylum- and class-specific primers for real-time PCR quantification of bacterial taxa. *J Microbiol Methods*. (2011) 86(3), 351-356. doi:<https://doi.org/10.1016/j.mimet.2011.06.010>

5. X. Guo, X. Xia, R. Tang, J. Zhou, H. Zhao and K. Wang. Development of a real-time PCR method for Firmicutes and Bacteroidetes in faeces and its application to quantify intestinal population of obese and lean pigs. *Lett Appl Microbiol.*  (2008) 47(5), 367-73. doi:10.1111/j.1472-765X.2008.02408.x

6. Y. Yang, M. Chen, B. Yang, X. Huang, X. Zhang, L. He, et al. Use of 16S rRNA Gene-Targeted Group-Specific Primers for Real-Time PCR Analysis of Predominant Bacteria in Mouse Feces. *Appl Environ Microbiol*. (2015) 81(19), 6749-56. doi:10.1128/aem.01906-15

7. J. R. Thompson, M. A. Randa, L. A. Marcelino, A. Tomita-Mitchell, E. Lim and M. F. Polz. Diversity and dynamics of a north atlantic coastal *Vibrio* community. *Appl Environ Microbiol*. (2004) 70(7), 4103-10. doi:10.1128/aem.70.7.4103-4110.2004

8. J. A. Frank, C. I. Reich, S. Sharma, J. S. Weisbaum, B. A. Wilson and G. J. Olsen. Critical evaluation of two primers commonly used for amplification of bacterial 16S rRNA genes. *Appl Environ Microbiol*. (2008)74(8), 2461-2470. doi:10.1128/AEM.02272-07

9. L. Mølbak, K. Klitgaard, T. K. Jensen, M. Fossi and M. Boye. Identification of a novel, invasive, not-yet-cultivated Treponema sp. in the large intestine of pigs by PCR amplification of the 16S rRNA gene. *J Clin Microbiol*. (2006) 44(12), 4537-40. doi:10.1128/jcm.01537-06
