# Supplementary material for: Host Defense Effectors Expressed by Hemocytes Shape the Bacterial Microbiota From the Scallop Hemolymph
Source: Front Immunol. 2020 Nov 12;11:599625. doi: 10.3389/fimmu.2020.599625 (PMC7689009; doi:10.3389/fimmu.2020.599625)
Supplement: Supplementary file 3 [file Table_2.docx]

| **Supplementary Table 2**  Bacterial strains used in this study to amplify 16S rDNA | |  |  |
| --- | --- | --- | --- |
| **Bacterial group** | **Bacterial strain** | **ID or origin** | **Donated by** |
| Firmicutes | *Staphylococcus aureus* | SG511 | Dr. Hans-Georg Sahl |
| Betaproteobacteria | *Alcaligenes sp.* | lab isolated | Dr. Michael Zeeger |
| Epsilonproteobacteria | *Helicobacter pylori* | lab isolated | Dr. Leda Guzmán |
| Gammaproteobacteria | *Escherichia coli* | ATCC 25922 |  |
| *Vibrio* spp. | *Vibrio splendidus* | VPAP18 | Dr. Rodrigo Rojas |
