# Supplementary material for: Host Defense Effectors Expressed by Hemocytes Shape the Bacterial Microbiota From the Scallop Hemolymph
Source: Front Immunol. 2020 Nov 12;11:599625. doi: 10.3389/fimmu.2020.599625 (PMC7689009; doi:10.3389/fimmu.2020.599625)
Supplement: Supplementary file 4 [file Table_3.docx]

| **Supplementary Table 3**  **Number of reads resulting from DADA2 process per sample** | | | | | |
| --- | --- | --- | --- | --- | --- |
| **Sample ID** | **Raw** | **Filtered** | **Denoised** | **Merged** | **Non-chimeric** |
| *NI-1* | 165779 | 127842 | 127842 | 127035 | 126360 |
| *NI-2* | 163000 | 125672 | 125672 | 124699 | 124067 |
| *NI-3* | 186269 | 146801 | 146801 | 140444 | 132187 |
| *NI-4* | 195528 | 152747 | 152747 | 151464 | 150048 |
| *NI-5* | 182560 | 141750 | 141750 | 139783 | 135782 |
| *SW48-1* | 167658 | 130309 | 130309 | 127704 | 119906 |
| *SW48-2* | 162529 | 127713 | 127713 | 126574 | 124731 |
| *SW48-3* | 171018 | 131939 | 131939 | 130871 | 129777 |
| *SW48-4* | 173368 | 135753 | 135753 | 135119 | 134370 |
| *SW48-5* | 146051 | 109933 | 109933 | 109187 | 108842 |
| *SW168-1* | 194950 | 151389 | 151389 | 150168 | 147197 |
| *SW168-2* | 154331 | 118298 | 118298 | 117650 | 116806 |
| *SW168-3* | 160899 | 121809 | 121809 | 121246 | 120288 |
| *SW168-4* | 188968 | 148369 | 148369 | 146739 | 144088 |
| *SW168-5* | 198318 | 153465 | 153465 | 150568 | 143402 |
| *VS48-1* | 179196 | 140342 | 140342 | 139432 | 138445 |
| *VS48-2* | 181756 | 134712 | 134712 | 132239 | 128578 |
| *VS48-3* | 149839 | 114787 | 114787 | 114170 | 114075 |
| *VS48-4* | 153919 | 117387 | 117387 | 116438 | 116087 |
| *VS48-5* | 171081 | 131742 | 131742 | 125318 | 110303 |
| *VS168-1* | 141728 | 110092 | 110092 | 109327 | 108983 |
| *VS168-2* | 153472 | 118969 | 118969 | 118071 | 117718 |
| *VS168-3* | 167275 | 128171 | 128171 | 127347 | 125428 |
| *VS168-4* | 165988 | 127258 | 127258 | 126136 | 124692 |
| *VS168-5* | 153834 | 119324 | 119324 | 118458 | 117807 |
